# Cultural and Religious Equity and Disparity in the Group Climate Experience of Moroccan Girls in Dutch Residential Youth Care Settings

**DOI:** 10.3390/ejihpe14060110

**Published:** 2024-06-08

**Authors:** Rabia Sevilir, Nienke Peters-Scheffer, Peer van der Helm, Dorien Graas, Robert Didden

**Affiliations:** 1 Department of Health and Welfare, Windesheim University of Applied Sciences, 8017 CA Zwolle, The Netherlands; r.sevilir@windesheim.nl (R.S.); tam.graas@windesheim.nl (D.G.); 2 Behavioural Science Institute, Radboud University, 6500 HE Nijmegen, The Netherlands; nienke.peters-scheffer@ru.nl; 3 Department of Forensic Child and Youthcare, University of Amsterdam, 1012 WP Amsterdam, The Netherlands; helm.vd.p@hsleiden.nl; 4 Expert Center Social Work and Applied Psychology, University of Applied Sciences Leiden, Zernikedreef 11, 2333 CK Leiden, The Netherlands; 5 Trajectum, Dokter Stolteweg 17, 8025 AV Zwolle, The Netherlands

**Keywords:** residential youth care, living group climate, Moroccan Dutch girls, cultural and religious equity, sense of belonging, identification, interpretative phenomenological analysis

## Abstract

Background: The group climate within residential youth care institutions is considered a transactional process, both within a group of youth from various cultural backgrounds and between them and their group workers. The ongoing interaction between the cultural characteristics of these girls may influence the quality of the group climate. This study aimed to provide an in-depth account of the living group climate experiences and perceptions of Dutch girls with a Moroccan cultural background in Dutch residential groups. Method: Interpretative phenomenological analysis was employed to explore the girls’ group climate experiences. Result: Three major themes emerged, namely (a) level of involvement of Moroccan girls in their living group, (b) perceptions of Moroccan girls’ sense of belonging in a living group, and (c) cultural and religious equality or disparity results in two interaction patterns. The findings revealed that equality or disparity in language, culture, and religion affect Moroccan girls’ experiences and perceptions of the living group climate. A crucial finding was that cultural and religious disparity leads to interaction patterns wherein the girls pre-emptively exclude themselves from receiving support from native group workers. Conclusion: Professionals must be aware of the cultural and religious dynamics, including the interplay and impact of cultural and religious equality and disparity, influencing Moroccan girls’ group climate experiences.

## 1. Introduction

In most European countries, including the Netherlands, the number of youths with different cultural backgrounds in residential youth care institutions (RYCIs) has increased [1,2]. Dutch youth with a non-Western cultural background constitute 18% of the total Dutch youth population. They are twice as likely to be in RYCIs compared with native Dutch youth, particularly in secure RYCIs. However, recent research has indicated differences in the utilisation of residential youth care (RYC) among the four main non-Western groups—namely Moroccan, Turkish, Surinamese, and Antillean. Specifically, youth with Moroccan and Turkish cultural backgrounds make less use of this form of youth care compared to those from Surinamese and Antillean backgrounds, with a usage rate similar to that of native Dutch youth [3]. This is noteworthy as Moroccan and Turkish groups have a similar high prevalence of emotional and behavioural problems, as well as a higher prevalence of similar risk factors. This raises questions about the accessibility and effectiveness of RYC for youth with a Moroccan or Turkish cultural background, which is concerning. In addition, these youths also have a higher risk of dropping out of RYC due to their cultural background and minority status, further increasing the risk of them not receiving sufficient care. Consequently, there is a heightened risk of overrepresentation of youth from certain cultural backgrounds in compulsory intensive youth care [4]. Dutch (noncorrectional) RYC assumes two forms: open (ORYC) or secure (SRYC; i.e., Youthcare^Plus^), each comprising several group homes [5].

Both of these public facilities offer 24 h group living facilities and provide care, pedagogical support, education, and treatment for youth between 0 to 23 years old with severe emotional and behavioural problems [6]. Concerning the level of restrictions, ORYC is considered the least restrictive form of youth care. While SRYC offers intensive care and treatment in a secure environment to youth who have been placed there coercively by a judge under a civil measure, ORYC offers treatment to youth who are voluntary, with their own or their parents’ consent, as well as under a civil measure [7,8]. The quality of the social environment in these institutions has a positive influence on the treatment and development of youth, as examined from the perspective of the living group climate. Stams and Van der Helm [9] (p. 4) have defined living group climate as “the quality of the social and physical environment in terms of the provision of sufficient and necessary conditions for physical and mental health, well-being, contact and personal growth of the residents. This is included with respect for their human dignity and human rights as well as, if not restricted by judicial measures, their personal autonomy, aimed at recovery and successful participation in society”. RYCIs monitor their living group climate using the validated Group Climate Inventory (GCI), a self-report questionnaire that measures the quality of the social environment from the perspective of residents [10]. Studies have demonstrated that the living group climate can be open or closed [10,11]. An open living group climate is characterised by responsive group workers, high levels of support and autonomy, possibilities for growth, a positive atmosphere, and low levels of repression, and in which youth and group workers treat each other with respect. A closed living group climate comprises low levels of support and autonomy and higher levels of repression. An open climate is conducive to the positive well-being of youth and is associated with improved social information processing, increased motivation for treatment enhanced prosocial behaviour, a decrease in treatment dropout, higher levels of internal locus of control, improved active coping, and increased empathy [12,13,14]. The living group climate is considered a transactional process, not only within a group of youth from a variety of (cultural) backgrounds but also between them and their group workers, the majority of whom are of Dutch origin [10,15]. There is a continuing interplay between the different characteristics of these youth, such as their cultural background, and those of the group workers; this vibrant interplay is associated with the quality of the living group climate [16,17]. Specifically, research has demonstrated that, in contrast to native Dutch youth, Dutch youth with a Moroccan cultural background (hereinafter “Moroccan youth”) experience lower levels of support in terms of a lack of trust, respect, and being taken seriously [18].

However, the literature has not provided insights into which personal perceptions and individual experiences underlie and influence the aforementioned outcomes. For example, how and to what extent the cultural characteristics of Moroccan youth, particularly girls, affect their living group climate experiences remain unknown. These girls face various challenges when they are placed in residential living group environments. Specifically, within such groups, these girls may encounter various cultural expectations that require them to navigate between two worlds. These girls’ upbringings impose clear rules and restrictions on them. These imposed rules and the collectivist values that stem from traditional or religious obligations, which are rooted in their upbringing within the Moroccan community in Dutch society, stand in contrast to the more individualistic approach they experience in Dutch living groups. This can lead to a clash of conflicting values, which may affect their living group experiences; thus, these girls may struggle with the intricate dynamics of cultural preservation and their adaptation to a more individualistic environment. Until now, no research has explored and provided an in-depth narration of the personal living group climate experiences of Moroccan girls in RYC.

Therefore, this study aimed to identify culturally grounded meanings of the concepts of support, growth, repression, and atmosphere in the context of these girls’ living group climate experience. The study was guided by the following research question: What experiences do Dutch girls with a Moroccan cultural background have in Dutch RYC regarding the particular living group factors of support, growth, repression, and atmosphere?

## 2. Materials and Methods

### 2.1. Design

The current study employed a qualitative design based on interpretative phenomenological analysis (IPA) [19], enabling a detailed exploration of Moroccan girls’ personal perceptions and individual experiences as well as of the living group climate phenomenon. Specifically, IPA was employed as a qualitative method for conducting a detailed exploration of how individual girls attach significance to their personal, intergenerational, and transnational social world in an individual manner [19]. IPA was consistent with the methodology of this research as it aimed to understand and describe the unique experiences of Moroccan girls, the meaning they attribute to them, and the significance they attach to their personal experiences, challenges, and needs. IPA allowed for a thorough examination of how these girls experience the living group climate in RYC. This was the most appropriate research method because this study’s focus was on obtaining information about personal experiences, the meaning attributed to them, and the participants’ own perspectives. No hypotheses were formulated due to its qualitative and exploratory nature [20].

### 2.2. Participants and Setting

Thirteen semi-structured interviews with Moroccan girls, group workers and a Muslim spiritual caregiver were conducted to explore how girls from Moroccan cultural backgrounds experience the climate of their living group. First, six Moroccan girls, 16 and 17 years old, were interviewed. The cultural background of the participants was deemed to be Moroccan if at least one of their grandparents or their parents had been born in Morocco. At the time of the study, all girls were living in three 24/7 RYCIs. All were born and raised in the multicultural Randstad area of the Netherlands, which spans The Hague, Amsterdam, Rotterdam, and Utrecht. In general, youth with Moroccan cultural backgrounds are often raised in socio-economically disadvantaged neighbourhoods within these urban areas [21].

These institutions provide care, education, and support to youth with identified emotional or behavioural needs in partnership with their families and aim to protect as well as prepare them for reintegration into society. Youth are living in groups of approximately 8–12 individuals and are supported by a team of trained group workers, which is who are responsible for the daily support of these youth. To contextualise the experiences of the girls and to obtain a more comprehensive representation of perceptions of the climate in the living groups, five Dutch group workers with Moroccan cultural backgrounds, one native Dutch group worker, and one Muslim spiritual caregiver were interviewed. Except for the native Dutch group worker, all participants identified themselves as Muslim.

### 2.3. Procedure and Data Collection

Group workers of the participating institutions were asked to recruit Dutch-speaking individuals aged 14–23 years, who resided in open and secure residential groups, and who had a Moroccan cultural background. The main reason for selecting the 14–23 years age group was the ability of youth to engage in (self-)reflection and share their personal perceptions and individual experiences. Participants were excluded if their pedagogical staff member determined that they would be unable to provide informed consent to be interviewed and/or participate in an interview. The group workers and spiritual caregivers were approached directly by the first author.

After the Ethics Committee of the Faculty of Social Sciences at Radboud University had approved the study (ECSW-2021-160), the authors provided information on data collection and the purpose of the study to all of the participants verbally and in writing. The participants were also assured of the anonymity and confidentiality of the data. During the interviews, the participants were informed and reminded that their participation would be voluntary and confidential and that they could withdraw from the study at any time. All participants gave their oral and written consent. After they agreed to participate, the participants were informed that they would receive a EUR 5 gift card as a token of appreciation for their contribution to the research.

The data were collected in line with the IPA method and through in-depth semi-structured interviews [22,23]. The interviews, conducted by the first author who had received the requisite training, were guided by a topic list with visual support about group climate. In accordance with the four subscales of the GCI (i.e., “support”, “growth”, “atmosphere”, and “repression”), interviews were guided by these four central topics, aligning with the respective subscales of the test. Initially, participants were asked to answer open-ended questions about the relative importance of the four topics for Moroccan girls. For example, the girls were asked, “If we look at these four factors of the living group climate, which topic do you find the most important?”. The group workers were asked, “If we look at these four factors of the living group climate, how do you think Moroccan girls experience support in the group?”. Then, the participants were encouraged to share their personal experiences. Every interview ended with the question, “Do you have any recommendations about what the living group climate for Moroccan girls should look like?”.

The interviews were conducted in an open and flexible way. The interviewer sought to initiate a dialogue with the respondents by following their trains of thought, and the topics from the list were treated as bases. The respondents were free to speak on other matters, and the discussion was open-ended because the participants’ perspectives and experiences were the cynosure of the study. The interviews were arranged so as to be private and convenient for the participants. They were conducted in a safe environment at the youth care centre. Each conversation lasted between 45 and 60 min. The anonymised interviews were recorded (with the informed consent of the participants) and transcribed verbatim by the first author for coding purposes. The audio recordings were deleted after transcription.

### 2.4. Data Analyses

In line with the IPA guidelines and following the stages set out by Smith et al. [19], the first Dutch author, who has a Turkish cultural background, and the second author, a native Dutch individual, conducted the analyses independently. The second author was not involved in data collection [19]. Furthermore, the first author was born in Turkey and has been living in the Netherlands since migrating as a young child. In addition, the first author identifies as Muslim, which is apparent to participants because she wears a headscarf. The first stage of analyses began with a thorough reading of the transcripts, enabling the authors to acquaint themselves with their content and to obtain a broad appreciation of the experience of the interviewees as they pertain to the subject matter of the study. Secondly, the authors read the transcripts line by line and noted points of descriptive, linguistic, and conceptual significance. Annotations were made about interesting remarks. Thirdly, the authors then reread the transcripts and their original notes, which enabled them to identify higher-level themes. Those themes were listed and examined. In the fourth stage, the themes that appeared to be related were grouped into superordinate themes and given descriptive labels. This process was repeated for each transcript. Once a connection between themes had been identified, the focus would shift to the next participant, and the themes that had emerged from the previous case were bracketed and included in the interview [19].

After the first author had conducted and transcribed the first two interviews, a meeting with the second author was held during which they reflected on the transcripts. The transcripts of the remaining interviews were also discussed extensively in periodic sessions. To ensure that the analysis was carried out in a rigorous way and that interpretations made by the first and second authors were of an explicit nature, a compare-and-contrast procedure was applied to each interview—emergent and superordinate themes were compared and discussed within the research team (the first, the second, and the last author) so as to identify similarities, discrepancies, and patterns across cases. Code saturation was reached after six interviews with Moroccan girls had been analysed; no new theoretical findings emerged from the subsequent coding and comparisons [24,25,26]. Following this, a decision was made to conduct interviews with group workers to gather contextual information. These interviews provided additional context and perspectives on the experiences of the Moroccan girls. Insights gleaned from the group workers helped contextualise the experiences of the Moroccan girls within a broader framework, including consideration of external factors that may have influenced them. Subsequently, a decision was made to conduct an interview with a provider of Islamic spiritual care to gain knowledge about the Muslim identity of the adolescents and its impact on their experiences of the climate of the living group. Meaning saturation was reached after six interviews with group workers and one Muslim spiritual caregiver had been analysed. No new themes, nuances, or insights emerged, indicating a comprehensive understanding of the issues at hand [24,25,26].

A critical consideration for this study was the complexity of the youth’s situation and the problems they encounter. Common problems faced by youth placed in RYCIs are also reinforced by cultural adaptational factors, including the youth’s process of migration, ethnic minority position, and cultural background. Moreover, age is a crucial factor considering that these youth’s ability to reflect sufficiently on their situation is not fully developed. Therefore, it was fundamental for the researcher to possess knowledge of these youth’s backgrounds. This knowledge enabled the researcher to put their situation into perspective, as well as to interpret how their backgrounds influence their interaction with the youth care setting and living group climate experience. The perspective of the researcher (first author) was also tested in these periodic sessions of reflection and analyses with the two other researchers (the second and the last author). The process also involved the interpretative activity of the researcher, otherwise known as the “double hermeneutic”.

The researcher’s impact on the context of a study is recognised in IPA. Accordingly, the authors acknowledge that their prior experience could have had an impact on the development of the themes. Transparency, internal coherence, and consistency were enhanced by means of double coding and through discussions of the emergent and the superordinate themes that were conducted within the research team [27]. This approach, taken in its totality, reduced the prior experience bias of the researchers, which would have otherwise resulted in unwarranted assumptions and prejudices being reflected in the content of the study. Ultimately, the overall rigour and trustworthiness were improved, and the credibility and conformability of the data were enhanced.

## 3. Results

Three themes emerged from the interviews in which the Moroccan girls reflected on the support that they had received from their group workers and on their relationships with their group mates. The first theme concerns the degree of the Moroccan girls’ engagement in the groups and their preferences for interpersonal interactions within them, as well as the factors that influence these two variables. The second theme pertains to the Moroccan girls’ perceptions of belonging to a group. The contours of those interpersonal interactions are outlined in order to define a sense of belonging. The last theme pertains to the effect of a common cultural heritage, Islamic identity, and minority status in Dutch society on the Moroccan girls’ interaction patterns.

### 3.1. Theme 1: Level of Involvement of Moroccan Girls in Their Living Group

In the interviews, all the girls distinguish between two types of groups: those with an Islamic and cultural orientation and those with a Dutch orientation. According to them, an Islamic and culturally diverse group is characterised by a team that consists mainly of Moroccan or Muslim workers who offer support to young individuals from different cultural backgrounds who identify themselves as Muslims. Islamic and cultural values, traditions, and practises are central to these groups. The Dutch-oriented groups were described as being served by teams that mainly consist of white native Dutch workers, in which the environment is (implicitly) characterised by Dutch values, traditions, and practices.

All of the girls exhibited a preference for living in an Islamic and culturally oriented group. In contrast to their engagement in Dutch groups, the level of engagement in Islamic and culturally oriented groups was high among the Moroccan girls. For example, one girl said, “Here [in cultural and Islam-oriented groups], group workers understand me, but there [at Dutch institutions], you do not have that; you do not have that in most Dutch institutions. Nothing happens there. You are not helped; you just live there […]. I really think it is really difficult to place Muslim youth in a Dutch institution anyway. Because really, nothing matches you at all… When you take a Muslim child out of a home situation because you want to save the home situation, it is not wise or sensible to choose to place Muslim children in Christian institutions. Then, it is wise to take time and effort for the child and find a good place for her… If there were Islamic institutions for Moroccan youth, that would really be better for them, better than Dutch institutions” (6Y).

### 3.2. Craving for Equality in Identity in Their Interpersonal Interactions

All of the girls exhibited a preference for living in an Islamic and culturally oriented group with Moroccan or Muslim workers and Moroccan group mates because they craved cultural, linguistic, and religious connection as well as equality in identity, which they experienced as a sense of belonging. Respondent 2Y shared the following experience: “There is one group worker that I really like. The rest are also good, but she is the one I like the most. She is Moroccan, just like me. She knows where I come from. She has known me for a long time. She was always there for me when I had problems. She understands me, and she knows my Moroccan background” (2Y).

When the Moroccan girls spoke about their experiences, they reflected on their relationships with both group workers and group mates. In the Islamic and culturally oriented groups, the Moroccan girls were positive about the relationships that they had formed with their group workers. Even so, the Moroccan girls reported experiencing comfort, ease, a sense of trust, affiliation, and confidence when their group workers and their group mates had similar migration backgrounds, religious and cultural beliefs, values, and practises. The Moroccan girls preferred working with Moroccan or Muslim group workers because they understood their religious and cultural preferences and took them into account when providing day-to-day support and advice. For example, Respondent 6 said that Moroccan or Muslim workers who operate in a predominantly Islam-oriented environment prepare food in line with Islamic rules. The girls’ emotional connections were fuelled by their shared identity, which evoked feelings of mutual understanding. This created a sense of unity. The mutual bonds and warmth were such that they preferred group mates from the same geographical and cultural backgrounds, both in urban areas in the Netherlands and Morocco. Respondents 1 and 4 reflected on their sense of unity in terms of “sharing a common geographical and cultural background,” which they associated with an identical sense of humour, appearance, and dress style.

The craving for connection through cultural belief was also evident from the praise that strong collectivist attitudes towards group workers and group mates received. Moroccan girls, raised in a collectivist home culture, prioritise the interests of Moroccan group mates over their own, just as they favour the interests of their community and family over their own. One girl said: “Look, we were one team, one task, and even if you got yourself into trouble, you were not going to betray your girlfriend—that’s not honourable. I didn’t have that with the Dutch because they were like, ‘No, I’m not going to get myself into trouble’. As Moroccans, we had a lot to offer [to] each other. Moroccan teams would never betray each other and would always rank their groupmate first. Regardless… even if you get into trouble yourself” (6Y). Although the girls had been born and raised in the Netherlands, they felt more Moroccan than Dutch. They all described themselves as Moroccan: “I’m just Moroccan, and that’s where I feel most at home. I do not feel Dutch at all, not really” (4Y). Attachment to Moroccan culture was strong among the girls.

The Moroccan girls mentioned that a certain culture had to be prevalent among their group mates for the atmosphere to be positive. Like most Moroccans in the Netherlands, these girls had grown up in the four large cities of the multicultural Randstad conurbations: Amsterdam, Rotterdam, The Hague and Utrecht [21]. The girls reported that they spent most of their leisure time with mixed-culture groups of peers who had grown up in these urban areas. All Moroccan girls reported that they sought each other out in the living group, as they had done in their previous family homes. While Respondent 1Y attributed the pleasant atmosphere to the group mates’ willingness to behave openly as a result of equality in growing up in a multicultural city, Respondent 5Y attributed it to mutual understanding through the use of identical language, the adoption of identical cultural beliefs, and reliance on identical codes of conduct: “I have the most connection with the Moroccan girl’. We speak the same language and may thus communicate secretly… Our culture, taboos, and parenting styles are also the same. We also share the same humanity within the same community. So, everything just clicks. Then, we understand each other far better than anyone else” (5Y).

Although the girls were fluent in Dutch, they still preferred interaction with bilingual group workers and group mates who spoke and understood both their mother tongue and Dutch. The Moroccan girls indicated that they felt comfortable and relaxed in an environment in which they did not have to adapt or consider the language in which they communicated: “I usually speak half Dutch and half Moroccan. While having a conversation, I make certain comments either in Moroccan or in Dutch, and if a Dutch group worker supports the group, we may just get punished; they see it as gossiping in Moroccan, but in the presence of a Moroccan group worker, it is never a problem” (5Y). The use of religious and cultural phrases and exclamations, such as “Inshallah!” (“In God’s will!”), the greeting “As-salamu alaykum!” (“Peace be upon you!”) and “Sabir” (“to be patient”), which are used by Muslim group workers and group mates, including Moroccan ones, had a positive influence on relationships. According to Respondent 5Y, the use of such words created a bond between them.

The girls rarely mixed with their Dutch group mates. They would only socialise with Dutch group mates whom they saw as not “cheesy” (The girls repeatedly associated the concept of being “cheesy” with a Dutch identity, irrespective of the individual’s original cultural background. They linked the use of the term “cheesy” to identifying individuals as exhibiting “Dutchness”, which refers to those who exhibit typical Dutch habits in their behaviours, actions, and thinking, and who generally have limited interaction with people with other cultural backgrounds. The girls predominantly perceived being “cheesy” as negative, which led them to avoid anything they considered cheesy).

According to the girls, young individuals who have interacted more frequently with Moroccan or Muslim youth are less “cheesy” due to their connections to multicultural urban areas. Moreover, these young “less cheesy” individuals had also modified their ways of life to accommodate the culture of the Moroccan girls. One girl perceived her Dutch girlfriend, whom she did not see as particularly “cheesy”, as Moroccan: “I also regard her as more Moroccan than Dutch. If you are less cheesed, you have grown up surrounded by more immigrants. You grew up with us. Then, you have learned more about the immigrant world than they have about their native culture. She is familiar with and understands our culture, what can and cannot be done, our taboos, and how to react to specific people” (5Y).

### 3.3. Craving for Family Feelings in Their Interpersonal Relationships

The importance of family was a subject that came up often during the interviews. For the girls, receiving support from a group worker with whom they could identify culturally, religiously, and linguistically was akin to receiving support from a family member. One girl said, “[H]ere, group workers are kind of family to each other. I was told that this was deliberately chosen so that we feel more at home….she [Moroccan group worker] told me, ‘you belong to the family now’… and that is really nice… It felt just like home. ‘The group workers cook, and there is always food when you get home… You can come and just eat. And when you are not present in the group, there is always food waiting for you, just like at home” (3Y).

These identifications created an atmosphere that resembled a family setting; they described their group workers and also their group mates by using words such as “sister/brother” or “cousin”. The emotional connections that the girls formed with the Moroccan or Muslim group workers and group mates were similar to those that they formed with caring family members, and the support was reportedly closer, frank and personal. One girl said, “He really is a kind of brother figure. He gives advice like a brother. A Dutch group worker could not be a brother figure; even if he is involved, then he is a ‘sir’. My group worker is also involved, but he is also Moroccan, and he is from the same city in Morocco” (5Y). These feelings of emotional proximity influenced the girls’ behaviour towards Moroccan or Muslim group workers: they expressed their feelings more openly, they were more respectful, and they behaved more politely towards them. The girls indicated that being polite and respectful towards others are important values in a Moroccan upbringing. However, they associated these values with being ashamed of the mainly Moroccan Muslim group workers, whom they saw as similar to older brothers or sisters.

The group workers also thought that Moroccan girls had more respect for and felt more shame around the Moroccan or Muslim care workers because they identified with them and saw them as family members. A group worker reported the following: “What I often hear from young people, for example, is that there is more respect for us from them than for my colleagues with a Dutch background… I see that in practice as well… These youth think, ‘I am now supported by them, as I was used to at home until now’. That feels the most comfortable and the most recognisable… From the youth, I heard, ‘you are my big brother’. Well, by being a big brother, you belong to their ‘we’, and you already earn respect on that basis” (2G). The Moroccan group workers also saw the girls as family members and exhibited a stronger willingness to provide additional support, which was highly valued, as also reflected by Respondent 6G. In addition, the importance of emotional connections that resemble family bonds for the group workers is also evident from the manner in which those connections are arranged by Islamic and culturally oriented institutions, as highlighted above in response 3Y.

### 3.4. Theme 2: Perceptions of Moroccan Girls’ Sense of Belonging in a Living Group

The Moroccan girls saw Moroccan and Muslim group workers and group mates as being more involved in social interactions and in the provision of support than native Dutch ones. The social interactions between Moroccan or Muslim group mates, as well as interactions between them and their group workers, were linked by the girls to a “home atmosphere”. These experiences, coexisting with equality in the Islamic cultural frame of reference, were associated with a sense of belonging to a group. The following themes characterised the girls’ perceptions of this involvement and belonging: “being interested in me”, “helping me”, “approaching me spontaneously and informally”, “conversing with me”, “paying more attention to me and being understanding”, “having a cosier relationship with me”, “having mutual respect”, and “striving for group consensus and interdependence between groupmates”. Respondent 3 Y remarked the following on the themes that characterise girls’ perceptions of involvement and belonging; “The Moroccan Muslim group workers do fun things with you. What you do really fascinates them, and you really see that these people care more about you than they do at… Here, it is really pure, from their hearts; they really care about you. Look, if I don’t have bread, they get me bread, while I don’t even have it on my shopping list… On the other living group [with Dutch staff members], they simply did their job, not even adequately or properly, and left. It was as if they were only there for the money. They were always in their office and never came over to check on you or inquire how you were” (3Y).

The girls described “interest” by using phrases such as “doing things with you together and caring about you”. While the theme “helping me” was associated with practical, emotional, cultural, and religious support, one of the girls reported receiving practical support: “Recently, I have been having a lawsuit. Then they joined me. They have helped me a lot. In the case of school, they also figure out what is best for me. They also know what I want and how I can succeed. Also, they help me with other things, such as arranging and reminding me of appointments” (3Y). The Moroccan girls allowed themselves to be emotionally supported by Moroccan or Muslim group workers despite not being used to expressing their feelings at home. One girl’s experience was representative of this tendency: “showing emotions… That was not something we did at home… At school, I cried and felt really badly…. And I’m actually not allowed to go outside without two group workers… They [the Muslim group workers] arranged everything to walk with me, so I felt better. Then, I thought, ‘see, they just thinking about me when I feel bad’. That feels good” (1Y). The girls connected their feeling of being emotionally supported to their experiences of benefiting from more consideration, understanding, and empathy. Involvement in support was also associated with sensitivity to the girls’ cultural and Islamic backgrounds. The girls indicated that the group workers supported them in managing issues that are common in collectivist cultures, such as dealing with shame, as reported by Respondent 5Y.

Spontaneous approaches in the group and informal conversation were perceived as positive aspects of the support that the Moroccan or Muslim group workers provided. Respondent 2Y described spontaneous inquiries into her feelings and needs as follows: “If I feel sad, they approach me and have a deep conversation with me. If I am sad, then they come to me and ask me how I am doing, how they can help me, and what I need from them. This is good support for me. In that other [Dutch-oriented] group, the support was zero. You are just put in a room there, and there is no one talking to you. You are not asked how you are doing. You are just in the group and sitting. No one comes to you from how you are” (2Y). The planned conservations with predominantly Dutch group workers in the Dutch-oriented groups were perceived less positively.

A positive atmosphere in which the girls respected each other was also considered important for their sense of belonging, as reported by Respondent 2Y using phrases such as “because you live together, 24 h a day you hang out with each other”, with Respondent 6Y associating mutual respect with respect for cultural customs, such as traditional dress. She reported on a situation in which she felt disrespected by her Dutch group mate: “I was wearing Moroccan pyjamas, and then I came downstairs to grab something… the Dutch groupmate said something like, are you in I.S.? [Islamic State terrorism]’. Look, you really should not do that kind of thing to me. How you want to wear your pants with pink flowers or whether you want to walk naked on the street at 20 degrees doesn’t matter to me; do what you want to do!” (6Y).

In addition, the Moroccan girls prioritised group consensus and thus strived to maintain unity, cohesiveness, and connectedness within the living group. According to one girl, “In order to establish a homey atmosphere, it is vital that we all have fun and feel one together, not just with the girls but also with the group workers. On the open group [that consists mainly of Moroccan or Muslim girls], we really lived together. Every day, we were together. Everything was done in collaboration. We had a good time wherever we went. We told each other everything. All of us were one. For that, we need to be able to trust each other” (1Y). Maintaining group cohesion was also associated with the theme of being strong as an in-group as reported by Respondent 6Y.

Furthermore, girls also mentioned interdependence, which, like group consensus, was thought to be a component of collectivism that inheres in the culture of the girls’ communities. Mutual assistance in times of stress, the sharing of personal information, and lending goods were among the recurring themes, which is evident from the following example: “We were basically in each other’s rooms every day. We went to borrow each other’s goods. I assisted her in getting dressed. She borrowed my makeup” (3Y). In addition, the Moroccan girls saw conviviality and mutual interest as being critical for both a positive atmosphere and for support from the living group workers. For example, one girl said, “You can laugh together and find humour in each other. You can make it cosy; you are not dull…I just arrived at the living group, and the girl with a Surinamese cultural background quickly came up to give me a big hug. This could have occurred on the other group, but not with as much intensity as she did, unless it was my Turkish or Moroccan girlfriend” (3Y). A positive atmosphere was described as entailing warm, pleasant, and enjoyable interactions.

### 3.5. Theme 3: Cultural and Religious Equality or Disparity Results in Two Interaction Patterns

Cultural and religious equality or disparity resulted in two distinct interaction patterns. On one hand, all the Moroccan girls exhibited proactive openness, honesty, respect, and trust towards their Moroccan or Islamic group workers. On the other hand, they excluded themselves from receiving support from native Dutch group workers in advance by reacting less openly and consciously becoming more distant. Nonetheless, these girls valued openness about all matters, including their own culture and domestic situation, in their collaboration. This openness was predominantly shown in their interactions with Moroccan or Muslim group workers. The group workers also thought that the Moroccan girls were craving connection through cultural and religious equality in the provision of support, which affected their mutual interaction patterns: “the same background and Islamic values… which allows you to understand certain choices of theirs much more, much faster, or to understand certain thought patterns of theirs… than someone who is Dutch…many Turkish and/or Moroccan group workers do not want to work on Friday because of the Friday congregational prayer; it is an important day [in Islam]. Muslim youth know and understand why these group workers do not want to work. I also hear them say, ‘Oh, tomorrow is Friday!’… Moroccan youth let go of things much faster; they are often more open and honest in certain matters that they [do] not share quickly with Dutch group workers” (2G).

As shown above, group workers took the view that Moroccan youth tend to choose mentors from a Moroccan or Muslim background because they are looking for recognition of their Islamic or cultural identity and because the provision of support can account for their beliefs. This example also shows that both Moroccan group workers and Moroccan girls prefer to discuss culture and religion openly and honestly with each other. The girls said that cooperation based on openness about all matters, including one’s own culture and domestic situation, was important: “Being honest, not lying to each other, and being transparent… and that is present here at the group… that you may readily communicate with each other and feel safe doing so. Therefore, I need trust. When I say something to them, it sticks with them” (2G). The girls appeared to be concerned about their reputation, which is why it was more important for them to trust each other. This example also shows the importance of trustworthy and understanding peers. According to the Moroccan group workers, cultural or religious identity engenders trust in both Moroccan girls and their parents. One group worker reported: “Parents think it is a Turkish or Moroccan group worker who knows what their religion and culture are like. This is also beamed to their children; that positively influences their trust toward us… Loyalty to their parents, culture, and religion is very high among these youth. If I can make the cultural and religious aspects of my support translate, and I also think I can make it better [than at my Dutch college], then they feel more understood” (1G).

### 3.6. The Influence of a Comprehensive Muslim Identity on the Moroccan Girls’ Daily Interactions

Moroccan girls, all identifying as Muslim, exhibit a profound attachment to Islam, influencing their attitudes and behaviours in daily interactions within group contexts. Notably, all Moroccan group workers also identified themselves as Muslim, whereas the native Dutch group worker did not. According to a spiritual carer, the Muslim girls in the living group experienced Islam as their core identity, which had an influence on the other roles that they assumed: “I carried out an identity task, so-called pizza slices. Each slice, named by the youth, represented their identity or role. The size of the slice meant how important the chosen identity or role was for them… What struck me is that the youth walked in the circle and formed one more central circle, their Muslim identity, which has an impact on all other identities and roles” (7S). The group workers unanimously made similar reports and added that engaging in conversations about religion can be a basis for setting goals, as reported by Respondent 6G: “Above all, I notice that religion is very unifying. Even after that, the country of origin is discussed… I have had a lot of antisocial youth who did not want to make any changes in their behaviour. If a strongly responsive Moroccan or Turkish colleague joins the group, then a sensitive chord is struck… It really goes back to religion and country of origin. There is a lot of talk about everything that is in the Koran: what makes someone a good person, when you are allowed to go to Allah, what it contains, and what it looks like… Moroccan and Turkish youth want to grow much more as human beings to be good people” (6G).

The requirements of Islam are considered important, and they have a direct or indirect impact on the girl’s attitudes, behaviours, experiences, and expectations about day-to-day support. For example, they adopt certain diets (halal food and non-alcoholic drinks), they need modesty and spirituality, they adopt certain dress codes, they fast during Ramadan, they celebrate its end, and they participate in the Friday congregational prayer. Modesty is valued in Islamic culture, which was reflected in the responses of the Moroccan girls. They highlighted specific issues that are related to modesty. The Moroccan girls had noticed that Muslim group workers were more aware of the notion of modesty in appearance and in the provision of support. One girl said, “[P]iercing, when I came to my mother asking if I could get a piercing, the answer would be ‘no’, and her reason would be that it is like a whore. While with Dutch group workers, it was like, ‘Oh, it looks beautiful’, while I had to hide my piercing from my mother. They are just so different. At…[Islamic and culturally oriented groups], for example, they also like it, but they understand why it was not allowed by my mother. Another example is if I have a deep cut in my shirt, then a Muslim worker comes to me, and she pulls on my shirt and tries to close it a little” (3Y). The girls were also aware of the need to behave modestly among Muslim group workers due to their upbringing. For example, they would not expose certain parts of their bodies to male group workers. Respondent 6Y reported that male Muslim group workers took this tendency into account by asking permission before entering their bedrooms so that they would have enough time to cover themselves up, as required by their dress code. Asking for permission before entering bedrooms is also important for Dutch girls, but the difference lies in the value that is ascribed to the practice, which may reflect privacy concerns or a feeling of shame. The Moroccan group workers indicated that their Dutch colleagues can and are willing to take this difference into account. The main difference is that Moroccan girls do not have to explain their concept of modesty to Muslim group workers. Group worker 2G reported that the girls preferred to expend less effort on explanations. She had had similar experiences: “If there is a new or substitute Dutch group worker, then the youth have to use words to explain it all over again… The youth often don’t like that… These youth also find it really difficult to explain and use good words for this” (2G).

Religious spirituality also shapes the Islamic identity of girls, their parents, and Muslim group workers, which enables them to bond and affects the support that they need in their daily lives. A spiritual carer stated that the girls’ need for behavioural change and development went hand in hand with their need for spirituality: “When they come to me, the most spiritual question is central: ‘So, how can I make sure I get satisfaction from my Creator? How can I be a good person? How can I be a good Muslim… What can I do? For example, should I read and get to know the Quran more… Or should I also learn the ethical part, so I can get along well with others?” (7S). According to the girls and the workers, parents are satisfied when their children are supported spiritually in an environment in which fear of Allah (taqwa, that is, doing good and staying away from evil) is present. A group worker shared the following: “If parents come here knowing that I am Muslim and I know what Islam is, then, in their eyes, I also know what fear of God is. You have a fear of God, and that has a certain meaning. You know that your child is being supported in an environment where there is also fear of God” (1G). Both the girls and the group workers were sensitive to spiritual conversations about hope (the reward of Allah), fear (of His punishment), and love (for Allah). These conversations strengthened their emotional bonds. Being Muslim herself, group worker 5G reported that her religion enabled her to meet this need easily. Responses to spiritual questions by Muslim group workers not only had a positive impact on relationships but also improved the well-being of the girls. According to the spiritual carer, “The [Dutch] group workers said, ‘You have done so many bath things... Why do you think it is important whether meat is halal or not… That has made the youth a little doubtful…made them feel inferior or bad Muslim… and made them ashamed of their Creator…Then, your living group environment, your group worker, made the difference… You have [a] group worker who said, ‘Fine, you should know yourself, this is your choice’, but you also have Muslim group workers who try to encourage, and they said, ‘Indeed, you have certain things that you also regret, and that is good. But it is no reason to distance yourself from your Islamic values or from your religious experience” (7S).

### 3.7. The Influence of Sharing the Same Life Experiences on Moroccan Girl’s Interaction Patterns

In the search for a balance between conformism and autonomy in the daily provision of support, the girls emphasised the importance of sharing the same life experiences with the group workers who had the same cultural background or Islamic identity and had grown up as members of a minority in Dutch society. One girl said, “My mother has a quite… pretty strict upbringing because she does not want her daughter to be talked about badly…Our community can really ruin your life just by gossiping about you. If the people with us see you as a whore outside, then you are also a whore to your family, while it is only seen… Then, Dutch group workers tell us to do what we prefer in this situation. And then, I realise, I cannot talk openly with my mother… You cannot talk about everything… I also believe being open is important, but being open in my community is also taboo… And then, I hear advice, and I have no clue what I can do with it… You really do not learn anything from them [native Dutch group workers]… You live together in a group, and you get advice, but really, we cannot do anything with the advice because we have a lot of taboos in our culture. You get advice, but you cannot apply it at home. In addition, our living environment consists only of our family. … maybe we can apply it at school or something with the Dutch teachers…They [Moroccan and Muslim group worker] understand how things are going with Moroccans. They see the same things I see” (5Y).

Although the Moroccan girls had been brought up to adopt collectivistic cultural values such as conformism, obedience, and respect, Dutch society encourages exploration outside of the home, and the support with which the girls are provided focuses mainly on autonomy and self-development. One girl reported on her struggle: “My mother would not allow me to wear short and tight clothes in summer… Finally, I left home, and I could put on whatever I wanted, and that was my choice. Nobody [at the Dutch-oriented institution] said anything about it. However, I noticed that it did not make me feel comfortable” (3Y).

The Moroccan girls indicated that they needed support that was aligned with both their specific needs and expectations and their Islamic and cultural frames of reference. The girls were searching for a balance between conformism and autonomy in the provision of support. Stable and protective support was essential for them to meet the expectations of their parents and the community, to maintain the honour of their families, and to be good Muslims. At the same time, they need support for their explorative behaviour that does not collide with their cultural and Islamic values. Respondent 3Y and Respondent 5Y (above) reported on their need for support, which was based on both conformism and autonomy. According to the girls, Dutch group workers struggled to provide such support. Conversely, group workers who had the same cultural background or the same Islamic identity and had grown up as members of a minority in Dutch society were more understanding of their situation due to having the same life experiences. According to the girls, similarity in life experience enabled those workers to provide advice that was more closely aligned to their specific needs and expectations and to their Islamic cultural frame of reference. Respondent 5Y (above) eventually also came to associate dissimilar life experiences with limited learning and unrealistic advice.

Support for bridging cultural contradictions was, in addition to parenting, regularly associated with the theme that covers relationships between the sexes. In Moroccan culture, interactions with the opposite sex are generally regulated by numerous rules. Girls, in particular, have to be careful not to violate these rules in courtship or in friendly relationships with boys. Although the girls were afraid of being detected by their parents and of gossip in the community, they would often secretly explore those aspects of life outside of it: “For example, boys… for Dutch group workers, it is very normal to take your boyfriend home and show it. They do not understand my upbringing at all. With us Moroccans that is just ‘No! Ready? You cannot have a boyfriend’. The Moroccan group workers told me that it used to be even stricter and that they had also experienced this. They told me that they were not allowed to talk to boys in the past, and you cannot just take a boy home from this, my friend” (3Y). The importance of being understood and supported in the context of cultural values, such as those that pertain to interpersonal relations and dependence, was once again emphasised with this theme. The strengthening of Muslim cultural identity that occurs as a consequence of common life experiences and understanding is the cornerstone of trust in group workers and their support.

The Moroccan group workers also indicated that they would serve as a bridge between the exploratory experiences of the Moroccan girls and the expectations of their parents as well as those of Dutch society. One group worker described this function as follows: “We, as a multicultural team, can make better translation between parents and children, compared to a group with mainly only Dutch workers: for example, the parents say this, but mean this; you, as a child, react [like] this, but you want to react like this.… With my experiences, my life experience, I can make the translation better and earlier, and am I able to take my Dutch colleagues here in the group by reflecting together? I was also brought up in that kind of situation with those norms and values, received my education here, and also experienced the same kind of situations. I do strive to be a kind of bridge for them to be able to make that translation” (1G).

Both the group workers and the girls associated common life experiences and tailored advice with the theme of finding the stay at the institution meaningful. According to the group workers, it is, therefore, necessary to create an environment in which the support is adapted both to the visions of the Dutch care system and to the religious and cultural practises, beliefs, and values of the girls. In providing pedagogical care, they were still seeking a balance between the Dutch individualistic values of the institution and the collectivistic Islamic culture that the girls had inherited from their home environments. A group worker reported that their support was closely aligned with cultural and religious frames of reference. An important condition here is that the measures that are adopted do not run counter to the methods of the institution: “[W]e cannot radiate certain norms and values to the youth… I cannot say to a girl, ‘Are you completely wise? You are not going to sleep with a boy anyway’. I do have certain lines of thought from my faith, but I can’t convey that directly…We work with methodologies in which girls can make their own choices… In my support, I am always looking for a balance… [H]ere, we are focused on the youth… Youth have a lot of rights; they can make their own choices, and this can clash with those genes that the parents want and find important… At a certain age, youth become wiser and realise that their parents were right… I made big mistakes. Because she was allowed to make those choices here in the group, and those choices clash very much with the cultural Islamic values, and I would really like to protect them in that because I really think that, after a certain age, they get more loyalty to these values and regret the choices they have made” (4G).

### 3.8. The Influence of No Confidence in Support from Native Group Workers in Advance on Moroccan Girl’s Interaction Patterns

On the one hand, the Moroccan girls reported that they had hardly received any support from the native Dutch group workers in the Dutch-oriented group, as represented by the following quote, which describes a common occurrence: “Dutch care workers were always in their office and never came over to check on you or inquire how you were” (3Y). On the other hand, if Moroccan girls knew that a Dutch group worker would be supporting the group, they would exclude themselves from the provision of support before the arrival of the Dutch group worker by reacting differently and by becoming more distant in order, according to them, to prevent further misunderstanding. One girl described how she and her Moroccan group mates excluded themselves from receiving support from native group workers in advance as follows: “We always have to deal with the group workers. So, always, when we know that a Dutch group worker has been scheduled to come to support, we think, ‘Oh, no, no, we are not going to talk today, we are not Ing to do fun today’… Because, yes, if we react in a certain way, they do not understand us. Then they think we mean it differently or in a different way. Then suddenly it is negative for them because we say it in a different way. You would better be quiet, then you will not get any nagging…I really had a lot of stomach aches at night; I could not really do anything, and I was really crying. The Dutch group worker, who also happened to be my mentor, opened my door in the night. But when I saw her, I just stopped crying. I was really in a lot of pain. I just stopped crying because I thought I couldn’t cry with you. That was the worst of all” (5Y).

The girls, as well as their parents, have certain prejudices against the provision of support by native Dutch group workers. The girls did not share their experiences of parenting and their (familiar) problems with the native group workers because they assumed that their domestic cultural circumstances and religion would be misunderstood due to a lack of familiarity. Moreover, they also assumed that they or their families would be disadvantaged or penalised for failing to judge situations in accordance with Dutch culture and to work in line with Dutch guidelines. According to the girls, the lack of understanding has to do with differences in life experiences and situations. This difference is seen as an obstacle to the provision of support and is thought to be impossible to eliminate through the acquisition of knowledge: “Dutch people have learned how to support us from a book. They do not know what it is like to be in such a situation… It is really a lot of little things happening at home that we understand in that context, and Dutch people just will not understand that. As much as they want to, they will never understand that. You cannot learn that from a book; you just have to have seen that” (6Y).

The responsiveness of group workers is partly determined by the behaviour of the girls. Therefore, it is difficult for Dutch group workers to provide sensitive support, as shown by their reports, which indicate that the Moroccan girls gave them little or no space to build trusting relationships. A Dutch group worker would often be required to expend more time and energy to gain their trust relative to a Muslim group worker. This tendency was reinforced by the lack of diversity among the youth and the group workers. One group worker said: “What strikes me is that if there are too many youth with no Dutch cultural background, then a Dutch group worker is already 3–0 behind [compared to Muslim group workers]… Then, the Dutch group workers enter into [a] discussion to reach a close compromise with them, but these girls are often not used to that, and they do not accept the authority of the Dutch group worker. That will be a difficult service” (4G). The Moroccan girls regularly reported events in which similar advice from the Dutch group workers was received negatively, even though it was a subtle behaviour that the Dutch group workers did not display. For example, while Moroccan girls wanted structure and both Moroccan and Dutch group workers strove to structure Moroccan girls’ days, the girls frequently associated it negatively with “strictness and overzealous adherence to regulations” by Dutch group workers, and positively with “helping me and protecting me above all from the prevailing taboos in Moroccan society” by Moroccan group workers. Respondent 3Y shared the following contradictory experience: “[B]eing at [Dutch-oriented] group to eat for six hours. I am aware it is typical of the Dutch. But no, I am not going to join the [Dutch-oriented] ‘group for dinner at six o’clock… They [native Dutch group workers] don’t provide structure in my life, they don’t care about how I’m doing…. Still, I believe that being on time for the group is an important rule at this institution [Islamic and culturally oriented groups]. In comparison to my previous [Dutch-oriented] group, they [Moroccan or Muslim group workers] have provided more structure for my life here” (3Y).

The group worker reported that enrolment into RYCIs and past negative experiences with Dutch authorities say at family guardianship institutions or within the youth care system as a whole, had also had a negative influence on preconceptions about support from native Dutch group workers: “Most youth have already experienced a lot outside, for example, with police or youth protectors, which is for all negative people, negative experiences. That’s all ‘white’… They are already entering with those prejudices… they associated Dutch group workers with them and think, ‘you are not going to do anything for me because you are just like them” (3G). The Western appearance of groups, as highlighted by Respondent 5Y in phrases “[T]hat was really a Dutch house… all herbs I had never seen before” (5Y), also amplified this tendency.

## 4. Discussion

This qualitative study established an in-depth account of the experiences and perceptions of Moroccan Dutch girls regarding the group climate in open and secure residential groups. To the best of our knowledge, this is the first detailed interpretative phenomenological account of such experiences. From the interviews regarding the particular living group factors of support, growth, repression, and atmosphere, the following three major themes emerged: (a) level of involvement of Moroccan girls in their living group, (b) perceptions of Moroccan girls’ sense of belonging in a living group, and (c) cultural and religious equality or disparity results in two interaction patterns.

The findings revealed that equality or disparity in language, culture, and religion affect Moroccan girls’ experiences and perceptions of the living group climate. Moroccan girls are a minority group in Dutch society. They have a strong desire to be supported by Muslim in-group workers as well as to live in an Islamic and culturally oriented group with group mates from the same cultural backgrounds and geographical places, such as urban areas in the Netherlands and regional areas in Morocco. Huijnk and Andriessen’s [28] research indicates that Moroccan youth, especially girls, show a strong preference towards their own cultural heritage. They frequently spend their free time engaging in social interactions within their own cultural community [29,30]. In this study, Moroccan girls’ interpersonal interactions were found to be characterised by their cravings for linguistic, cultural, and religious connection; equality in identity; and familial feelings, which they experienced as a sense of belonging. This need for connection through equality appeared to influence not just the Moroccan girls’ involvement in the groups but also their interaction patterns and interpersonal relationships within them. A crucial finding was that cultural and religious disparity results in interaction patterns in which the girls exclude themselves from receiving support from native group workers in advance by reacting differently and consciously becoming more distant. While they prevent further misunderstandings, this could ultimately hinder the establishment of a working relationship with the Dutch group workers.

Moreover, the Moroccan girls expressed a strong willingness to create connectedness through equality in culture and religion, which is probably based on their desire to keep their Moroccan home culture, Islamic religious heritage, and Moroccan and Muslim identity within the living groups [29]. Noteworthily, Moroccan girls seem to be able to identify better with group workers and members when the sociocultural distance becomes smaller, which appears to enter into interpersonal relationships and result in a sense of belonging to a group—the most significant component of ethnic identification [31]. Belonging involves shared connections and identifications with certain individuals or groups, along with the unique and distinct characteristics that differentiate us from others [32]. Furthermore, the girls appear to place the highest priority on their Moroccan identity, followed by their identity linked to their city of residency in the multicultural Randstad conurbations; above all, they prioritise their Muslim identity, which is in line with previous studies [28,33,34]. The girls’ strong identification with Islamic and Moroccan culture within the groups seems to be an identification process, where they search for connection in their interpersonal interactions based on reciprocity in terms of belonging to the same cultural or religious group, mutual identification, shared cultural and religious beliefs, common knowledge, behavioural codes, and patterns [35,36,37]. This seems to result in interaction patterns with identical modes of thoughts and expectations, a feeling of mutual understanding and trust, and an emerging feeling of unity.

There may be several explanations for the Moroccan girls experiencing their Moroccan and Muslim cultural identity as their core identity in their interpersonal interactions in the living group. First, these girls are in their adolescent stage, which is characterised by the formation of their identity, wherein exploration and commitment play important roles [30]. The exploration entails experimentation with different alternative directions and beliefs, while commitment involves the choices that an adolescent makes from several alternative paths in various fields, such as relationships, culture, and religion [30]. In the context of the Dutch culturally and religiously diverse society, identity development includes the search for and discovery of what it means to be a member of and belong to a particular cultural and religious group [31,38]. This search seems to be conducted within the living groups the girls reside in, where they think about their individual (problem) situations and circumstances in relation to their own personal wishes, desires, and needs. They also harmonise these considerations with the values that drive their cultural and religious backgrounds in a predominantly white, non-Islamic, Dutch and sometimes even anti-Islamic environment. In addition, the methods and protocols generally reflect Western institutional culture. These girls may experience contradictions between the norms and values inherited from their collectivist-oriented Islamic home cultures, which emphasise the connectedness aspect of identity, and the norms and values prevalent in a more individualist-oriented non-Islamic Dutch residential environment. When they choose to prioritise aspects of Dutch culture in their interpersonal interactions, they may experience a sense of neglect or denial of their Moroccan culture [35,39]. However, avoiding support from group workers with a different cultural background to their own could be deemed a missed opportunity. Dutch group workers possess the potential to offer fresh new perspectives to these girls, thereby familiarising them with alternative norms, values, and behavioural patterns [40].

Second, Moroccan girls may feel limited in their freedom and possibilities for exploration. This is because of traditions that view behaving collectively to be important and upbringing to have value based on honour, respect, conformity, obedience, good manners, and specific gender roles compared with individual growth [35,39]. These collectivist-oriented norms and values, which originate from the home country, seem to be especially strongly held by members of second and third generations [41,42]. Moreover, numerous Moroccan parents believe that adolescence is not a stage for experimentation and are anxious that their child will become too “Dutch” [35]. It is therefore questionable to what extent these girls feel freedom of choice to be able and allowed to experiment with alternate directions and beliefs by opening themselves up for native Dutch group workers’ support. Such workers have a more individualistic cultural frame of reference that values autonomy, independence, self-development, and determination. Therefore, the girls may naturally and/or unconsciously bond with group workers with a Moroccan cultural and/or Muslim background, which is in accordance with their parents’ requirements, personal wishes, and probably also expectations.

Third, this study identified a profound attachment to Islam among Moroccan girls, which influences their attitudes and behaviours in their daily interactions within group contexts. This finding aligns with previous studies that have indicated that among Dutch youth from Moroccan cultural backgrounds, Muslim identity frequently operates as a prominent and all-encompassing aspect of their identity, e.g., [43,44,45,46]. This deep-seated connection is rooted in the religious norms and values inherited from their religious upbringing, which they seem to regard as essential guidance for their lifestyle and daily routines within the groups. Despite differences in the degree to which the girls practice their religion, their Muslim identity is still considered an important guideline in their daily interactions [35,47]. Adhering to Islamic norms, rituals, traditions, and customs can significantly affect their daily life in residential living groups. These girls seem to be in search of ways to incorporate personal interpretations of Islamic norms and values into their daily lives in a living environment where they do not always seem self-evident. This search may lead to the desire for recognition and understanding to better shape their faith as well as to the need for support from Muslim group workers to bring their faith more in line with their behaviour. Moreover, Dutch group workers might exhibit reluctance to broach spirituality, religiosity, and religious coping in their contact with these girls [48]. Remarkably, this quest seems to be fuelled by the anti-religious or even anti-Islamic sentiments in residential care groups, which are often rooted in secular values. The Netherlands is among the most secular countries globally [49]. The girls may perceive this to be a threat, which might prompt them to cling more firmly to their religious identity. Conversely, this quest may be strengthened within culturally Islamic groups, where Muslim youths can support and strengthen each other in this spiritual journey. In either situation, this search process may lead to a form of segregation.

Fourth, Moroccan girls seek group workers and group mates with shared experiences and whose lifestyles and environments are similar to their own. This is because the girls at the institutions require support in bridging cultural differences and seeking a balance between their own personal wishes, conformism, and autonomy in the provision of support. The cultures in which these girls are participating are not only different but also, in certain aspects, contradict each other [50]. The Islamic cultural frame of reference of Moroccan girls as well as that of their Moroccan group workers and group mates have been shaped by the following comparable experiences: cultural adaptation issues, relative social and economic deprivation, commonly lower living conditions, and an upbringing in a collectivist-oriented home culture with strong Islamic influences as a member of the more individualistic, marginal religious influence-oriented Dutch society [29,41,42,51]. However, Moroccan youth also have a Dutch individualistic identity because of Dutch society’s encouragement of exploration outside of their home, autonomy, and self-development [50]. These girls, who are in the process of acculturation, experience certain tensions when they encounter situations that are difficult to resolve either by referring to the collectivistic norms of their culture and of their Islamic upbringing or by referring to the Dutch individualistic support [29,37]. Commonalities in life experience appear to allow Moroccan or Muslim group workers to serve as role models and assume a connective function as a cultural bridge; thus, they can provide support that is more closely aligned with Moroccan girls’ own personal wishes, needs, and expectations, as well as both their individualistic and Islamic collectivistic cultural context [30,52]. These group workers’ connective functions can possibly prevent marginalisation and stimulate social and individual success.

Fifth, the dominance of the Muslim and Moroccan identity is probably more pronounced in living groups that mainly consist of Moroccan and/or Muslim group workers and group mates. This is due to the themes, interests, and problems shared by youth with a Moroccan cultural background [29,37]. Adolescents have a strong desire to belong to a group and not be an outsider [29,30,31]. This wish seems to be fulfilled for the Moroccan girls, as they feel a part of their cultural or religious group by sharing a living group with group workers and group mates with the same partial identity and with whom they can talk about mutual similarities. In addition, this may lead to the assumption that the group workers and group mates will behave in accordance with their own personal wishes and also with their parents’ and community’s expectations and needs, which could ultimately create a positive feeling. Moreover, these group workers could possibly connect better with the girls’ world and may be more sensitive, particularly to otherness, because of optimal interfaces such as overlapping preferences, views, interests, topics of conversation, a sense of humour, activities, and life stories, which seem to create understanding and respect as well as to positively influence the self-confidence and self-esteem of youth [52]. By contrast, the efforts made by Dutch group workers to understand the girls’ world through questioning may probably be experienced as unnatural by the girls and could cause them to perceive being “the other”.

Another explanation for the pronounced cultural and religious profiling of Moroccan girls within the living groups could be associated with them entering the RYCIs with negative interpersonal experiences, such as (social) rejection, exclusion, and (institutional) discrimination [33,37]. When the girls in these living groups face implicit or explicit experiences of exclusion and encounter unconscious displays of anti-immigration or anti-religious sentiments, stressing their Islamic identity can actually provide a positive sense of security, stability, and belonging [37,53]. Choosing their Muslim identity within the groups allows them to feel part of the Islamic “ummah” (i.e., the community of Muslims), instead of relating to a negative perception of being an “outsider” [50,54,55]. This can result in strong mutual cohesion with Moroccan and Muslim group workers and group mates; however, it can also result in a dismissive attitude towards Dutch non-Muslim group workers and members. This strong “we–they” dynamic can influence the responsiveness of the native Dutch group workers, potentially leading to reduced availability and involvement within the group. This mutual influence arises from a transactional process between group workers and youth [15]. Furthermore, this “we–they” dynamic is also apparent in the girls standing up for each other as Muslims and their emotional connection with Moroccan or Muslim group workers and group mates, which resembles a family bond. The Moroccan culture also places high importance on family and local community ties. This may all account for the Moroccan or Muslim group workers’ stronger willingness to provide additional support to the girls, as well as for the girls’ more polite, open, and respectful behaviours towards Moroccan or Muslim group workers. According to Hendriks et al. [52], the shared cultural or religious background and common experiences provide distinct tools for social workers, fostering a heightened sensitivity to otherness and to other norms and values, thereby enhancing their understanding of and support for their clients.

## 5. Limitations

The findings of this study should be cautiously interpreted in light of several limitations. The first limitation is the study’s predominant focus on qualitative information derived from a small group of respondents, which makes generalising the results difficult. This is the first qualitative study to have explored Moroccan Muslim girls’ experiences of the living group climate. The aim of capturing these experiences through an examination of how these girls experience the aforementioned climate in RYCIs was not to generalise but rather to create an in-depth, contextualised narration of these personal living group climate experiences. Second, the Moroccan girls were from a single, geographically diverse area of the Netherlands, namely the multicultural Randstad area, which encompasses The Hague, Amsterdam, Rotterdam, and Utrecht. Just under half of all residents of Moroccan origin live in one of these four major cities (CBS). Thus, the question arises as to whether the same results would hold true in regions that are generally less multicultural than these four major cities. Future research should therefore consider replicating this study in regions outside of the Randstad area to ascertain whether the observed findings remain consistent in less culturally diverse areas.

## 6. Implications and Future Research

RYCIs seek to create an open living group climate for youth—regardless of their cultural background—that responds to their basic psychological needs for relatedness, competence, and autonomy [56]. However, understanding the living group climate from Moroccan girls’ perspective poses significant challenges to group workers. Establishing partnerships with the cultural context of the youth is an element of high-quality care in RYCIs [2]. Particular challenges for Dutch group workers involve the establishment of trusting relationships, a critical factor in the living group climate. The main indications of effective relationships are group workers’ ability to use positive treatment, such as providing sufficient contact, ensuring positive communication, and being respectful to the youth. Group workers should be able to use their support skills regardless of the youth’s specific cultural backgrounds. A concern arises when Moroccan girls pre-emptively exclude themselves from contact and support, which hinders the development of trusting relationships.

Mutual understanding, empathy, and awareness of cultural and religious values, beliefs, and rituals are crucial, not only in the interactions between group workers and youth but also in fostering positive relationships among the youth within a living group and creating a positive learning environment. Both Moroccan girls and group workers need to incorporate understanding and awareness of as well as empathy for each other’s cultural and religious values, norms, and beliefs. If the obstacles to establishing trusting relationships between Moroccan girls and native Dutch group workers are to be overcome and the specific needs of these girls are to be responded to, culturally sensitive behaviours and attitudes in interpersonal interactions are required from both parties. Thus, RYCIs should provide heightened support to supervise these girls in their acculturation adjustment process by emphasising the development of a bicultural identity within the groups. This support should enable the girls to feel a sense of belonging to both their cultural and religious groups as well as to the larger majority group in the living groups. Simultaneously, the disparities in their craving for connection during interpersonal interactions, based on equality, could be reduced by the practice of culturally sensitive care. Culturally sensitive care refers to support adapted to the specific wishes and needs of youth with different cultural backgrounds, and it is provided by culturally competent group workers. This need for cultural sensitivity has been emphasised in various studies [18,57,58]. However, the extent of cultural competence among Moroccan or Muslim group workers and native Dutch group workers remains an open question. While Moroccan or Muslim group workers might excel in building relationships due to a shared cultural Islamic frame of reference, comprehensive cultural competence is not automatically guaranteed.

This objective can be achieved through the development and implementation of cultural sensitivity that involves collaborative engagement between group workers and the youth, as well as among the youth themselves. This training will serve as a pivotal tool in establishing, augmenting, and enhancing cultural alignment, coherence, match, and cohesion between the youth and group workers as well as among the youth themselves. Structured group meetings conducted during these training sessions ought to be facilitated distinctly, bifurcating between sessions that involve the youth and group workers and sessions exclusively for group workers. The aim of these sessions is to openly delve into, discuss, and address topics that are intricately related to the cultural and religious diversity prevalent within the living groups.

A recurring subject that often came up during the interviews was the importance of Moroccan girls receiving support that aligns with their parents’ Islamic and cultural expectations as well as their satisfaction when the girls receive support in an Islamic culturally oriented group, particularly with Moroccan or Muslim group workers. Furthermore, parents’ Muslim identification has an impact on their children’s sense of being both Muslim and Moroccan [59]. Further research is required to investigate the extent to which and understand how the cultural Islamic frame of reference held by parents within a collectivist Islamic community may influence the living group climate experiences and perceptions of Moroccan girls within their living group environments. Moreover, considering the significant and all-encompassing role that religion plays in the identity of Moroccan girls and the impact of their strong attachment to Islam on their attitudes and behaviours in daily life as well as their living group experiences, researching how and in what manner spiritual and religious dimensions ought to factor into the support provided to living groups appears to be paramount.

## 7. Conclusions

This qualitative study offers valuable insights into the living group climate experiences of Moroccan girls in residential settings. It emphasises the necessity of professionals being cognizant of and understanding the intricate interplay and impact of equality and disparity in culture and religion on the Moroccan girls’ interpersonal interactions and their living group climate experiences. Their strong attachment to their Moroccan and Muslim identity leads to their desire to be supported by Muslim in-group workers and to reside in an Islamic and culturally aligned group with group mates from the same cultural backgrounds, which provides a sense of unity and belonging. However, concerns arise when differences in cultural and religious backgrounds lead to interaction patterns in which these girls exclude themselves from contact with and support from native Dutch group workers in advance. This exclusionary tendency impedes the development of trusting relationships; limits the exploration of diverse perspectives, values, and norms; and ultimately poses a risk to integration, potentially leading to segregation.

## Data Availability

Data is unavailable due to privacy.
